# Natural Antioxidants and Hydrocolloids as a Mitigation Strategy to Inhibit Advanced Glycation End Products (AGEs) and 5-Hydroxymethylfurfural (HMF) in Butter Cookies

**DOI:** 10.3390/foods11050657

**Published:** 2022-02-23

**Authors:** Huiyu Hu, Yuting Wang, Yousheng Huang, Yanpeng Yu, Mingyue Shen, Chang Li, Shaoping Nie, Mingyong Xie

**Affiliations:** 1 State Key Laboratory of Food Science and Technology, China-Canada Joint Lab of Food Science and Technology (Nanchang), Nanchang University, 235 Nanjing East Road, Nanchang 330047, China; ncuhhy@163.com (H.H.); ncuskwangyuting@163.com (Y.W.); yshuang0526@163.com (Y.H.); ncuyyp@163.com (Y.Y.); shenmingyue1107@ncu.edu.cn (M.S.); lichang@ncu.edu.cn (C.L.); spnie@ncu.edu.cn (S.N.); 2 Jiangxi Institute of Analysis and Testing, Nanchang 330029, China

**Keywords:** advanced glycation end products, 5-hydroxymethylfurfural, butter cookies, natural antioxidants, hydrocolloids

## Abstract

Maillard reaction during food processing contributes to the formation of some unpleasant heat-induced toxicants including advanced glycation end products (AGEs) and 5-hydroxymethylfurfural (HMF). The current study prepared butter cookies fortified with two dietary natural antioxidants (catechins and curcumin) and two dietary hydrocolloids (pectin and chitosan), and investigated their effects on formation of free N^ε^-(carboxymethyl)lysine (CML)/N^ε^-(carboxyethyl)lysine (CEL), protein-bound CML/CEL and HMF and on the sensory qualities of butter cookies. Meanwhile, three typical α-dicarbonyl compounds were also determined to identify possible correlations between α-dicarbonyl intermediates and formation of these harmful heat-induced products in butter cookies. Experimental data showed that catechin exhibited the strongest inhibitory effects on formation of AGEs and HMF, but its addition would impair the color and taste of cookies. On the other hand, chitosan was not so effective in inhibiting AGEs and HMF as compared to catechin, but its addition could increase the sensory qualities of butter cookies.

## 1. Introduction

Butter cookies are widely distributed food commodities, favored for their characteristic baking flavors, aromas and color which is directly related to the Maillard reaction during baking [1]. Baking is an indispensable step in the processing of butter cookies and the Maillard reaction is responsible for the brown color and organoleptic properties [2]. This complex process brings about texture, chemical and structure changes in the dough matrix, such as volume expansion, evaporation of moisture, development of porous structure, denaturation of proteins, starch gelatinisation, crust formation and browning [3]. Although consumer acceptance is improved during this thermal processing, a series of hazardous compounds are generated, such as advanced glycation end products (AGEs) and 5-hydroxymethylfurfural (HMF) [4,5,6]. The accumulation and circulation of dietary AGEs are related to the onset/progression of diabetic complications [7], which may promote oxidative stress, inflammation, and atherosclerosis [8], and HMF may induce mutagenic and genotoxic effects in human cells and promote colon and liver cancer in rats or mice [9]. Therefore, great attention has been directed toward the possible adverse effects of these heat-induced food toxicants over the past decade [10,11,12].

The recipes for butter cookies all included sugar, flour, and butter as major components, where the condensation of an amino residue of proteins and a carbonyl group of sugars lead to a complex cascade of consecutive and parallel reactions during baking, and it has been selected as a food model in a variety of researches regarding the formation kinetics of AGEs, HMF and α-dicarbonyl compounds [13,14,15]. N^ε^-(carboxymethyl)lysine (CML) is known as a well-established AGE marker in foods and in vivo for the occurrence and levels of AGEs because of its stability and abundance [16]. The condensation reaction between glucose and ε-amino group of lysine forms fructoselysine, an Amadori rearrangement product, which is oxidized to form CML. It can be also produced by an alternative route through a reaction of lysine with glyoxal (GO). N^ε^-(carboxyethyl)lysine (CEL), known as another major AGE, is a homologue of CML and is mainly formed by the reaction of methylglyoxal (MGO) and lysine. GO and MGO, as the typical α-dicarbonyl compounds, are commonly produced during lipid peroxidation, Maillard reaction and sugar degradation [7]. HMF, another kind of heat-induced toxicant, which is an indicative compound in the Maillard reaction and caramelization, is formed by dehydration of hexoses or Amadori products. Glucose and fructose can generate HMF via a Maillard reaction and caramelization to form a α-dicarbonyl intermediate, 3-deoxyglucosone (3-DG) [17].

A previous study has shown that CML is extensively formed in all categories of cookies, with 3.94 ± 0.09 mg kg^−1^ in butter cookies, 1.73 ± 0.60 mg kg^−1^ in wholemeal cookies, and 0.11 ± 0.04 mg kg^−1^ in dry cookies [18], whereas other have found CML and CEL contents in biscuits were 117.53 and 46.09 µg g^−1^ of biscuits, respectively [19]. Moreover, free AGEs and protein-bound AGEs in the real food system were found to have different bioavailability and physiological effects [20], yet there were only a few studies that determined both [21,22]; related research in butter cookies has been rarely reported. The formation of HMF is directly related to the heat load applied to many foods, sometimes exceeding 1 g kg^−1^ in certain dried fruits and caramel products [23]. Cookies using glucose and sucrose as raw materials at a baking temperature of 200 °C were found 18 ± 11 mg kg^−1^ and 5 ± 4 mg kg^−1^ of HMF, respectively [24], while 23.74 ± 0.08 mg kg^−1^ of HMF was detected in butter cookies [18].

Since the ubiquitous occurrence of AGEs and HMF, it is of great importance to mitigate AGEs and HMF formation in butter cookies. Fortunately, some mitigation strategies can be applied to inhibit their formation as indicated by the literature [12,15,25]. The best approaches appearing so far are involved in intervention in the reaction mechanisms. For example, the formation of AGEs from baking foods (biscuit, bread) was inhibited by the presence of natural antioxidants such as epicatechin and (+)-catechin acting as free radical scavengers [26,27]. Hydrocolloids such as chitosan have also been shown to exert inhibitory effects on Maillard reaction products, because chitosan contains amino groups which can react with the carbonyl groups of reducing sugars to form chitosan-sugar conjugates [28].

At present, simultaneously mitigating AGEs and HMF formation in butter cookies has never been taken into consideration, and the possibility of using a combination of natural antioxidants and hydrocolloids in the reduction of heat-induced toxicants in butter cookies has never been explored. In recent years, hydrocolloids have been used in some food systems to control the Maillard reaction; meanwhile, the efforts of chitosan on the microbial stability and staling behavior of the final product made utilization of chitosan in bakery recipes provides great advantages as well [29] and low molecular weight chitosan exerted a potent inhibitory effect on Maillard reaction products (>52.6%) [30]. 5% pectin was added in biscuits lead to a 67% decrease in acrylamide. Pectin are acidic polysaccharides, which can provide an acidity environment without significant interference in taste or color [31]. However, the potential impact of natural antioxidants and hydrocolloids in alleviating formation AGEs and HMF have rarely been studied.

The present study aims to use two dietary natural antioxidants (catechins and curcumin) and two dietary hydrocolloids (chitosan and pectin) to investigate their effects on the formation of AGEs and HMF and on the sensory qualities of butter cookies. The antiglycation potential of natural antioxidants and hydrocolloids in cookies was also assessed with reference to the formation of the reactive α-dicarbonyl intermediates. Moreover, it is well known that the selection of additives influences the visual appearance, color, flavor and therefore consumer acceptance, so a sensory evaluation and color values were carried out to figure out the acceptance of sensory attributes of cookies. This study hopes to provide a reliable strategy of controlling AGEs and HMF formation during cookie baking without impairing sensory properties, and different chemical and sensory indicators for assessing the quality of heat-processed foods are effective for the control of processes, and open up more possibilities for optimizing manufacturing conditions.

## 2. Materials and Methods

### 2.1. Chemical and Reagents

HMF, [U-^13^C_6_]-HMF, 3-DG, quinoxaline, 2-methylquinoxaline, o-phenylenediamine (OPD), 5-methylquinoxaline (5-MQ) and formic acid were obtained from Sigma-Aldrich (St. Louis, MO, USA). CML and CEL standards as well as d_4_-N^ε^-(carboxymethyl)lysine (d_4_-CML) and d_4_-N^ε^-(carboxyethyl)lysine (d_4_-CEL) standards were purchased from Toronto Research Chemicals Inc. (North York, ON, Canada). Ammonia solution (≥25% in H_2_O), monobasic and dibasic potassium phosphate were of HPLC grade and obtained from Shanghai Aladdin Biochemical Technology Co. (Shanghai, China). Analytical grade chloroform, sodium borohydride, sodium borate, boric acid, sodium hydroxide and hydrochloric acid were purchased from Sinopharm Chemical Reagent Co., Ltd. (Beijing, China). Methanol (MS grade) was acquired from Merck (Darmstadt, Germany). Dietary curcumin (≥90%), pectin (≥80%) and chitosan (≥90%) were purchased from Xingmu Biological Engineering Co., Ltd. (Shenzhen, China). Dietary catechins was purchased from Hongda Tea Co., Ltd. (Wuyuan, China), and the composition of catechins is summarized in Supplementary Materials (Table S1). Oasis HLB or MCX solid phase extraction cartridges (6 cc, 200 mg) were supplied by Waters (Milford, MA, USA). Deionized water was provided by the Watson Group Ltd. (Hongkong, China).

### 2.2. Preparation of Butter Cookies

Butter cookies were made from wheat flour (low gluten, recommended use for cookie making), butter, eggs and icing sugar (sucrose), which were purchased from local supermarkets. The cookie recipe consisted of wheat flour (80 g), whole egg (12 g), icing sugar (20 g), butter (20 g), and deionized water (2 g). The ingredients were thoroughly mixed with different concentrations (0.3%, 0.7%, 1%, 2%, 3%, 4% and 5%, *w*/*w* of dough) of natural antioxidants or/and hydrocolloids compounds by an electronic blender. The dough was rolled and placed into a mold to form a disk with a diameter of 5 cm and a thickness of 3 mm. Cookies were baked at 170 °C for 12 min in a digital oven operated without forced air circulation (CO-2701 model, COUSS Electric Co., Ltd., Zhongshan, China). Finally, the cookie samples were rapidly cooled to room temperature with ice, ground and kept at −20 °C until further analysis after baking. All experiments were performed in triplicate. The dietary natural antioxidants and hydrocolloids that added are dietary additives with Chinese food production license, and the amount used was permitted by Chinese law.

### 2.3. Analysis of Free CML and CEL, Protein-Bound CML and CEL, HMF and α-Dicarbonyl Compounds in Butter Cookies

#### 2.3.1. Sample Preparation for Analysis of Free CML and CEL, Protein-Bound CML and CEL and HMF in Butter Cookies

Ground cookies powder (0.5 g) were added to a 10-mL centrifuge tube along with 10 μL of d_4_-CML and d_4_-CEL mixed internal standard solution (10 mg mL^−1^, prepared in a 20:80 methanol/water solution, *v*/*v*) and 20 μL of ^13^C_6_-HMF internal standard solution (10 mg mL^−1^, prepared in a 20:80 methanol/water solution, *v*/*v*) and 2 mL of deionized water. Each sample was defatted twice using extractions of 4 mL of chloroform/methanol (2:1, *v*/*v*) solution [32], the mixtures were vortexed and centrifuged at 4400× *g* for 10 min to collect the supernatants and residue in the middle layer.

For free CML and CEL, supernatants were subjected to solid phase extraction (SPE) by using an automatic SPE instrument (PrepElite-GVS model, LabTech Co., Ltd., Beijing, China) equipped with Oasis MCX cartridge. The MCX cartridge was preconditioned with 3 mL of methanol followed by 3 mL of deionized water. Then 1 mL of supernatant was loaded onto the cartridge and washed with 1 mL of methanol and 1 mL of deionized water before being eluted with 4 mL of 5% ammonia solution in 80:20 methanol/water (*v*/*v*). To remove volatile ammonia, the eluant was concentrated gently with a stream of nitrogen gas to dryness, and resolved in 1 mL of deionized water, vortexed for 30 s, and then filtered through a 0.22 μm membrane before injection into the HPLC-QqQ-MS/MS system.

For protein-bound CML and CEL, the residue was transferred and dried completely with a stream of nitrogen gas in a 30-mL thick-wall pressure-resistant glass tube (Beijing Synthware Glass Instrument Co., Ltd., Beijing, China). Next, 1 mL 2 M NaBH_4_ (prepared in 0.1 M NaOH) and 2 mL of borate buffer (0.2 M, pH 9.2) were added and left overnight at 4 °C to proceed reduction. After reduction, 4 mL of 12 M HCl was added to the samples, and the pressure-resistant glass tube was flushed with nitrogen, sealed and incubated at 110 °C for 24 h. Next, the cooled acid hydrolysate was filtered through filter paper and diluted with deionized water to obtain a final volume of 25 mL. Finally, 500 μL of the diluted hydrolysate was evaporated to dryness with nitrogen gas (60 °C) and reconstituted in 1 mL of deionized water with the addition of d_4_-CML, d_4_-CEL and ^13^C_6_-HMF internal standard solutions for the same SPE clean-up procedure as mentioned above.

For HMF, 1 mL of supernatant was passed through an Oasis HLB cartridge preconditioned with 3 mL of methanol followed by 3 mL water. The column was washed with 1 mL of deionized water and finally eluted with 2 mL of methanol. The eluate was collected and filtered through a 0.22 μm membrane before injection into the HPLC-QqQ-MS/MS system.

#### 2.3.2. Sample Preparation for Analysis of α-Dicarbonyl Compounds in Butter Cookies

The levels of α-dicarbonyl compounds (GO, MGO, 3-DG) in butter cookies were analyzed based on steps described in previous studies with some modifications [13]. A 10 μL of 5-MQ (2 mg mL^−1^, internal standard) was added into 0.5 g of ground cookies powder before defatting as described above in Section 2.3.1; then 200 μL of supernatant was mixed with 400 μL of potassium phosphate buffer (0.1 M, pH 7.0) and 400 μL of OPD solution (1%, *w*/*v*) in a tube. After vortex mixing for 30 s, the tubes containing the reaction mixture were placed in a constant temperature incubator (SHP-150, Shanghai Sumsung Laboratory Instrument Co., Ltd., Shanghai, China) at 25 °C and kept in the darkness for 3 h. Finally, the sample solutions were transferred to autosampler vials after passing through a 0.22 μm syringe filter.

#### 2.3.3. HPLC-QqQ-MS/MS Analysis

The determination of free CML and CEL, protein-bound CML and CEL, HMF and α-dicarbonyl compounds in butter cookies was carried out on an Agilent 1290 Series high performance liquid chromatography system (HPLC, Agilent Technologies, Santa Clara, CA, USA) equipped with an Agilent 6460 triple-quadrupole mass spectrometer (Agilent Technologies Inc., Santa Clara, CA, USA).

The analysis of CML, CEL and HMF was performed on a Hydro-RP 80 Å column (150 mm × 2 mm, 4 μm, Phenomenex, Torrance, CA, USA) using isocratic mixture of methanol (A) and 0.1% (*v*/*v*) formic acid solution (B) at a flow rate of 0.2 mL min^−1^ at 30 °C, with 2 μL of injection volume. The gradient profile of the binary pump started at 99% B (0 min), was maintained to 3 min, and was then decreased to 80% B in 3 min; was then increased to 99% B over 2 min and finally, it was maintained at 99% B for 2 min for the purpose of equilibrating the column for the next injection. The electrospray ionization (ESI) source was operated in positive mode and selective detection using multiple reaction monitoring (MRM) mode was conducted. Quantitation was achieved by means of the internal standard method; the quantifying and qualifying transition ions were: CML (*m*/*z* 205 84, 130), d_4_-CML (*m*/*z* 209 → 88, 134), CEL (*m*/*z* 219 → 84, 130), d_4_-CEL (*m*/*z* 223 → 88, 134), HMF (*m*/*z* 127 → 109, 81) and ^13^C_6_-HMF (*m*/*z* 133 → 115, 86), the mass transition ions in bold was used for quantitation. The ionization parameters were set as follows: capillary voltage 3.5 KV; drying gas temperature: 350 °C; drying gas flow: 12 L/min; nebulizer: 35 psi; sheath gas temperature: 350 °C; sheath gas flow: 9 L/min; nozzle voltage: 500 V.

The analysis of quinoxaline derivatives of α-dicarbonyl compounds (GO, MGO and 3-DG) was accomplished on the same instrument and column mentioned above. The mobile phase consisted of 0.1% (*v*/*v*) aqueous formic acid solution (A) and methanol (B). The gradient profile of the binary pump was initiated at 60% A and decreased to 30% A from 0 to 4 min, then, held at 30% A from 4 to 6 min, and finally went back to 60% A in 2 min, and then equilibrating at 60% A for 2 min. The flow rate was 0.25 mL min^−1^, and the injection volume was 1.0 μL. The ESI source was operated in positive mode and the MS was performed in the MRM mode with the following settings: drying gas temperature: 300 °C; drying gas flow rate: 12 L/min; nebulizer pressure: 35 psi; capillary voltage: 4 KV. Quantitation was achieved by means of the internal standard method, the quantifying and qualifying transition ions were: GO (*m*/*z* 131 → 77, 51), MGO (*m*/*z* 145 → 77, 92), 3-DG (*m*/*z* 253 → 171, 199) and 5-MQ (*m*/*z* 145 → 91, 65), the mass transitions in bold was used for quantitation.

### 2.4. Measurement of the Color of Butter Cookies

The color of butter cookies was measured by the CIE L*a*b* system using a SC-10 portable colorimeter (Threenh Technology Co., Ltd., Shenzhen, China). Six measurements that are representative of the cookies were taken from each sample and averaged. In the color three-dimensional system, the coordinate L* indicates the lightness of color (the interval of 0–100 means black to diffuse white); a* characterizes the position between red and green (value range from −128 to 127, negative values indicate green, and positive values indicate red); b* characterizes the position between yellow and blue (value range from −128 to 127, negative values indicate blue, and positive values indicate yellow) [26].

### 2.5. Sensory Evaluation of Butter Cookies

The sensory panel consisted of ten panelists who were trained with the sensory profile method [33]. Five sensorial parameters were set up as taste, chewing, odor, color and appearance based on their liking value (V = [0,5], 5 = like very much, 0 = dislike very much). These sensorial parameters worth different weight coefficient (W), assigning taste value accounting for 40%, chewing value accounting for 25%, odor value accounting for 15%, color value and appearance value accounting for 10% respectively. Thus, the final total sensory evaluation value (S) follows the following formula:

(1) $$S=W\times V=[0.4,\;0.25,\;0.15,\;0.1,\;0.1]\times \left[\begin{array}{c} \begin{array}{c} \begin{array}{c} \mathrm{Taste}\;\mathrm{Value}\\ \mathrm{Chewing}\;\mathrm{Value} \end{array}\\ \mathrm{Odor}\;\mathrm{Value} \end{array}\\ \begin{array}{c} \mathrm{Color}\;\mathrm{Value}\\ \mathrm{Appearance}\;\mathrm{Value} \end{array} \end{array}\right]$$

### 2.6. Statistical Analysis

Experimental data were reported as mean ± standard deviation. All analytical determinations were carried out in triplicate. The significance of the difference was evaluated with one-way ANOVA (analysis of variance) and Duncan’s test (*p* < 0.05) by using SPSS Version 22.0. The Pearson correlation analysis was performed at a significance level of *p* < 0.05.

## 3. Results

### 3.1. Effects of Catechins and Curcumin on the Formation of AGEs and HMF in Buttter Cookies

**Figure 1:**
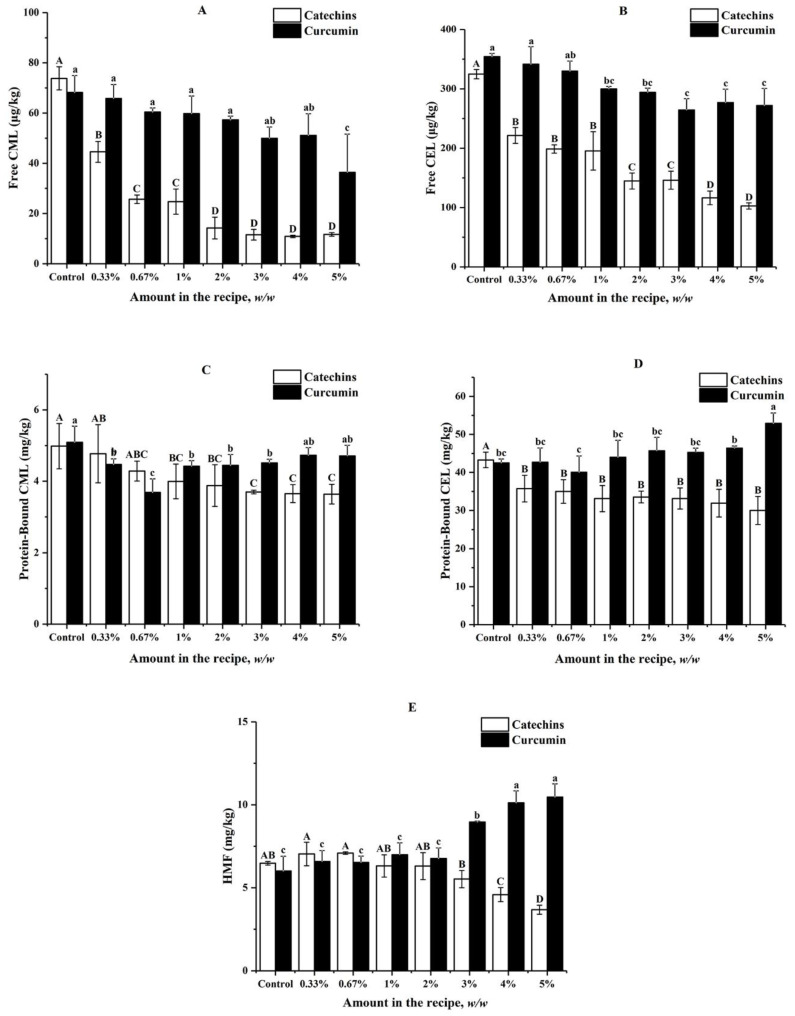
Impacts of different amount of catechins and curcumin in the cookie recipe on (**A**) Free CML; (**B**) Free CEL; (**C**) Protein-Bound CML; (**D**) Protein-Bound CEL; (**E**) HMF in cookies; values are expressed as mean ± SD (*n* = 3) for all treatments; different capital letters indicate significant differences (*p* < 0.05) among different addition level of catechins, while different lowercase letters indicate significant differences (*p* < 0.05) among different addition level of curcumin.

In this section, effects of catechins and curcumin on formation of free CML and CEL, protein-bound CML and CEL, and HMF in butter cookies were investigated (Figure 1). For control samples without the addition of catechins and curcumin, high levels of protein-bound CML (4.37–5.09 mg kg^−1^), CEL (42.48–50.79 mg kg^−1^), and HMF (5.08–6.48 mg kg^−1^) were observed; while extremely low levels of free CML (68.21–89.33 µg kg^−1^) and CEL (322.60–354.27 µg kg^−1^) were detected. Protein-bound CEL was the most predominant harmful product formed during baking of butter cookies, followed by HMF and protein-bound CML. These results were in consistent with those reported by Palermo [18], which presented for 3.94 mg kg^−1^ of CML in butter cookies and 4.73 mg kg^−1^ of HMF in wholewheat cookies.

As shown in Figure 1, the addition of catechins in butter cookies at concentrations ranging from 0.3% to 5% could inhibit free CML and CEL significantly, leading to 31.89–84.19% reduction and exhibiting a dose-effect relationship. Catechins could also inhibit protein-bound CEL significantly (15.32–30.64%), but a dose-effect relationship was not observed (*p* < 0.05). When catechins were spiked at levels between 1% and 5%, significant inhibitory effects could be observed for protein-bound CML (19.88–26.71%). However, no significant inhibitory effects could be observed for HMF until the spiking levels of catechins increased to 4% (29.17–43.21%).

The inhibitory effects of curcumin were not so excellent compared with those of catechins; for example, it could not inhibit free CML and CEL until the spiking level increased to 5% (46.62%) and 1% (15.35–25.34%) respectively. When curcumin was spiked at levels between 0.3% and 3%, significant inhibitory effects could be observed for protein-bound CML (11.39–27.70%). No positive or negative effects of curcumin at levels below 5% or 3% were observed for protein-bound CEL and HMF respectively; however, curcumin was found to promote formation of protein-bound CEL and HMF when spiked at the levels of 5% and above 2% respectively.

Catechins show an excellent inhibitory effect on CML and CEL formation at a low amount of addition, and as the level of catechin addition increases, the content of heat-induced toxicants (CML, CEL, HMF) in cookies decreases as well. Interestingly, it was found that catechins can often achieve an enough inhibition rate (65.25%) on AGEs at a low proportion (0.7%). In fact, Sylwia [27] concluded that the loss of phenolic compounds appears to be dependent on their chemical structures, which affect their thermal stability after baking. Epigallocatechin gallate (EGCG), accounting for 51.92% of catechins (Table S1) could reduce CML concentrations in a glucose–lysine model system mimicking different food processing conditions [34]. Our survey indicated that both catechins and curcumin have a significant inhibitory effect on free CML and free CEL than that of protein-bound CML and protein-bound CEL. Free AGEs are formed by the combinations between free amino acids and carbonyls, likewise; combinations between peptides/proteins and carbonyls produce protein-bound AGEs. Studies have shown that there are many differences between free and protein-bound AGEs: protein-bound AGEs in digestive tract could transit the epithelial cell and enter the circulatory system more efficiently than free AGEs and the renal clearance rate of protein-bound AGEs is lower than that of free AGEs, these variances indicate that free and protein-bound AGEs in food should have different influences on human health [20]. However, another review was found on this topic, AGEs of a different molecular weight may have different metabolic effects in the human body as well, Somoza [35] showed that up to 30% of low molecular weight Maillard reaction products (MRPs) or their degradation products in guts could be absorbed by the body; however, most of the high molecular weight MRPs were directly excreted in the form of faeces. Obviously, this excellent inhibitory effect of catechins on free AGEs, which are low molecular weight MRP, greatly reduces the probability of free AGEs being absorbed by the body. Curcumin did not have the same excellent suppression efficiency on both AGEs and HMF when added in the same amount. In particular, it had no significant effect on bound AGEs, and even a high proportion of curcumin (>3%) has a significant inhibitory effect on HMF.

### 3.2. Effects of Pectin and Chitosan on Formation of AGEs and HMF in Butter Cookies

**Figure 2:**
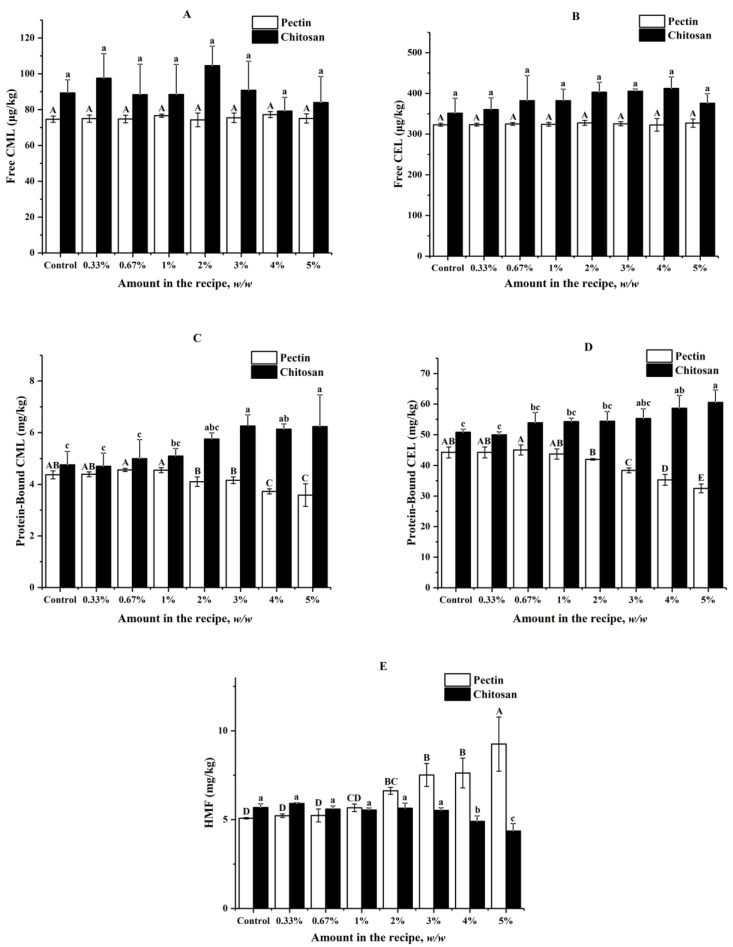
Impacts of different amount of pectin and chitosan in the cookie recipe on (**A**) Free CML; (**B**) Free CEL; (**C**) Protein-Bound CML; (**D**) Protein-Bound CEL; (**E**) HMF in cookies; values are expressed as mean ± SD (*n* = 3) for all treatments; different capital letters indicate significant differences (*p* < 0.05) among different addition level of pectin, while different lowercase letters indicate significant differences (*p* < 0.05) among different addition level of chitosan.

The impacts of pectin and chitosan on the formation of AGEs and HMF in butter cookies are shown in Figure 2, which suggested that both pectin and chitosan did not have statistically significant effects on formation of free CML and CEL at each spiking level (*p* < 0.05). Formation of protein-bound CML could be inhibited by pectin spiked at levels between 2% and 5% (6.18–18.08%), while could be promoted by chitosan spiked at levels above 2% (20.84–30.88%). Formation of protein-bound CEL could be inhibited by pectin spiked at levels between 3% and 5% (13.17–26.53%), while it could be promoted by chitosan spiked at levels above 3% (8.94–19.22%). However, no significant inhibitory effects could be observed for HMF until the spiking levels of chitosan increased to 4% (13.73–23.24%). Besides, the formation of HMF was stimulated by the addition of pectin (11.83–82.28%) at levels above 1% whilst it was inhibited by chitosan at levels above 3%.

As shown in Figure 2C,D, the two hydrocolloids did not bring significant effects on the free AGEs but high proportion of chitosan (>3%) brought about the promotion of protein-bound AGEs. Contrary to chitosan, pectin can effectively reduce protein-bound AGEs to a certain extent when the amount of pectin in cookie recipe is greater than 1%, but HMF increased remarkably when the addition amount of pectin was higher than 3%, which is probably due to the reduction of pH in the dough of cookies. As Supplementary Materials (Table S2) shows, when pectin added was higher than 2%, the pH value in cookies dough is significantly reduced, while chitosan has no obvious effect on the pH of the dough. It was reported that Maillard reaction undergoes mainly 1,2-enolisation with the formation of HMF under acidic or neutral conditions, at pH > 7 the degradation of the Amadori products is thought to primarily involve 2,3-enolisation, in which reductones and various fission products, including GO and MGO, are formed [36]. Obviously, pectin, known as an acidic polysaccharide, plays the role of acidity regulator in the cookie system.

Moreover, HMF significantly decreased when the concentration of chitosan was higher than 4%, which is consistent with the Mogol [29]. It was found that though the HMF formation was reduced by 34.70% and 40.20% in asparagine–glucose–chitosan model systems when heated for 20 and 30 min. In recent years, many researchers suggested that hydrocolloids such as pectin and chitosan added to the formulations of various heat-processed foods [31,37] had certain inhibitory effects on Maillard reaction harmful products such as acrylamide and HMF, or had the effect of improving taste, antibacterial and antifungal activities. It can be seen from our results that although pectin and chitosan have their own advantages in inhibiting AGEs or HMF, simultaneous control of AGEs and HMF formation by use of only hydrocolloids is not possible.

### 3.3. Effects of Natural Antioxidants and Hydrocolloids on the Formation of α-Dicarbonyl Compounds in Butter Cookies

α-Dicarbonyl compounds are important intermediate products originated from dehydration and fragmentation of Amadori or Heyns products, and they may also be formed by either sugar/lipid degradation [38]. GO, MGO and 3-DG are highly reactive α-dicarbonyl compounds capable of stimulating the Maillard reaction to form dietary AGEs after reaction with amino residues or even other intermediary compounds as HMF by dehydration [7].

**Figure 3:**
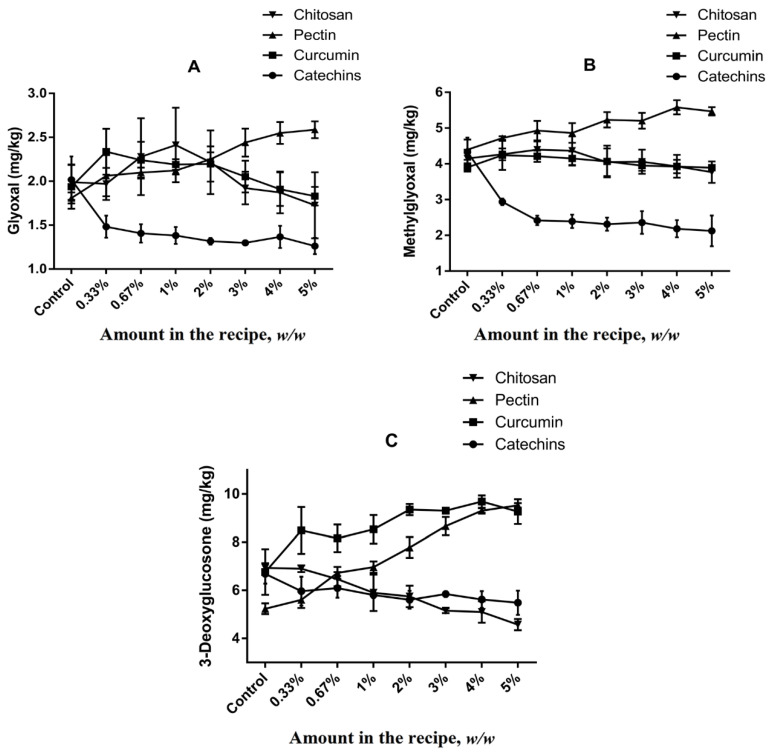
Impacts of different volume of addition on (**A**) GO; (**B**) MGO; (**C**) 3-DG in cookies. Values are expressed as mean ± SD (*n* = 3) for all treatments.

The formation of α-dicarbonyl compounds in different cookies models were studied (Figure 3). Levels of GO, MGO and 3-DG in cookies of control group were 2.02 ± 0.27 mg kg^−1^, 4.33 ± 0.40 mg kg^−1^ and 6.69 ± 0.41 mg kg^−1^, respectively, which suggested 3-DG was the most abundant α-dicarbonyl compound in butter cookies, followed by MGO and GO. Due to the high content of reducing sugars and low content of free amino acids in recipe of butter cookies, caramelization is considered to be the main pathway for the formation of α-dicarbonyl compounds during baking process [39]. 3-DG is the precursor of HMF which is the main product of the caramelization reaction, and GO and MGO are the precursors of CML and CEL in Maillard reaction, respectively [40]. It is verified from the experimental data that the higher content of precursor MGO than that of GO leads to a higher amount of CEL than that of CML (Figure 3).

As shown in Figure 3, catechins added at concentrations of 0.3–5% could inhibit the formation of α-dicarbonyl compounds in butter cookies, but no dose–effect relationships were observed (*p* < 0.05). The inhibitory effects of chitosan were only observed on 3-DG formation. Unfortunately, curcumin and pectin were not observed to have inhibitory effects on GO, MGO and 3-DG, and at certain spiking levels they even promote the formation of these α-dicarbonyl compounds, especially 3-DG. These results were expected and they are consistent with previously reported by Totlani [34,41], suggesting that catechins can traps α-dicarbonyls in a model system. According to the current research status, the inhibition mechanism of catechins can be classified into two categories: on the one hand, the antioxidant capacity and free radical scavenging capacity of catechins may play a role in inhibiting the formation of AGEs and HMF [42]. On the other hand, catechins may directly participate in the Maillard reaction, forming key intermediates with AGEs, such as adducts with active α-dicarbonyl compounds such as MGO or GO, thereby inhibiting the formation of corresponding heat-induced toxicants. Furthermore, Sun et al. [43] have suggested that the MGO-trapping characteristic of curcumin supports its inhibition of AGEs formation, but significant differences in MGO in the curcumin group of cookies was not observed compared with the control group in this study. Interestingly, it was found that dietary curcumin can reduce the GO content at high addition (>3%), which is in line with the ability of curcumin trapping capacity in other report [44]. Since pectin is an acidic polysaccharide (Table S2), Chen, X. M [45] found that 3-DG can be generated from a reducing sugar degradation product, and its formation is favored by acid-catalysis; moreover, our team also reported that a lower pH value resulted in more 3-DG generation in the model system [40].

**Figure 4:**
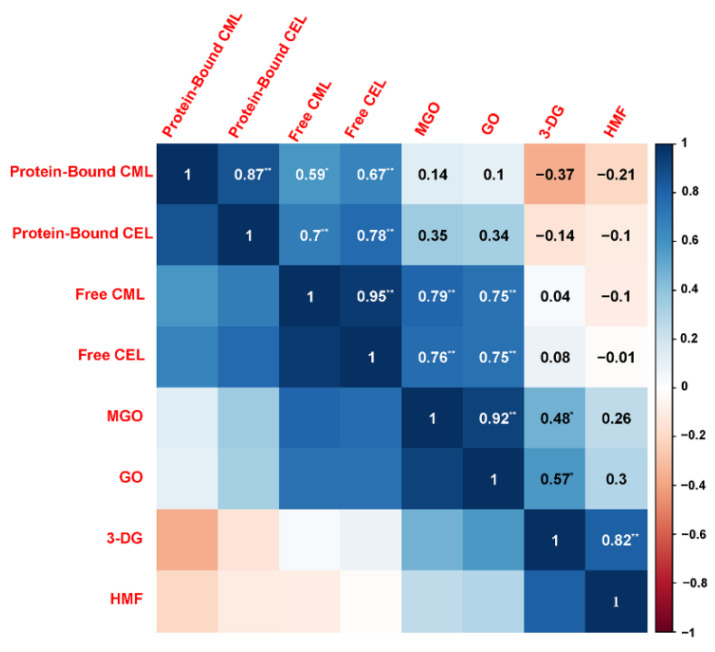
Pearson correlation analysis: the inhibition of protein-bound CML, protein-bound CML, free CML, free CEL, HMF and α-dicarbonyl compounds (GO, MGO, 3-DG) in cookies. Blue and red colors represented the correlation coefficients form 1 to −1. Asterisks marked significant differences (* means *p* < 0.05 and ** means *p* < 0.01).

The Maillard reaction in a real food system like butter cookies is obviously very complicated and too many variables in this model leads to inconsistent results. GO, MGO and 3-DG might regulate the Maillard reaction process and then affect the formation of some Maillard reaction-derived products, such as AGEs and HMF. The correlation analysis (Figure 4) according to Pearson’s correlation test was constructed to identify possible correlations between the various parameters changing in the cookies due to adding with catechins, curcumin, pectin, chitosan at different proportions, including free CML, free CEL, protein-bound CML, protein-bound CEL, HMF, and α-dicarbonyl compounds (GO, MGO and 3-DG).

Pearson correlation analysis showed that free CML/CEL and MGO/GO are extremely significantly correlated with correlation coefficients greater than 0.75, which were higher than those between MGO/GO and protein-bound CML/CEL. Meanwhile, HMF and 3-DG are extremely significantly correlated as well, with a correlation coefficient greater than 0.82. During the baking process, GO and MGO are fragment products produced by the retro-aldolization, which may be formed through a Maillard reaction, caramelization and lipid oxidation, while 3-DG is originated from the oxidative degradation of hexose. Due to the high content of reducing sugars in cookie recipes, caramelization is considered to be the dominant mechanism for the formation of 3-DG [39]. Although amino groups in chitosan can combine with the carbonyl groups of the reducing sugars to form MRPs, this study did not find enough evidence to prove that chitosan competes with amino acids or proteins for carbonyl groups.

### 3.4. Effects of Natural Antioxidants and Hydrocolloids on Color and Sensory Qualities of Butter Cookies

**Figure 5:**
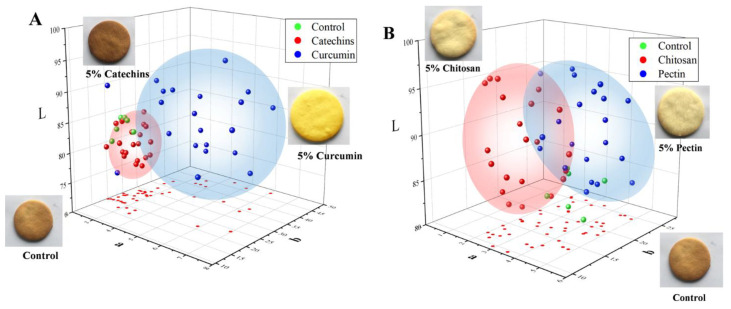
Confidence ellipsoid of dietary natural antioxidants and hydrocolloids com-pounds on the color of L*a*b* system in cookies. (**A**) Green sphere represented control cookies, red sphere represented catechins cookies, blue sphere represented cate-chins cookies. (**B**) Green sphere represented control cookies, red sphere represented chitosan cookies, blue sphere represented pectin cookies.

The color and sensory qualities were taken into consideration when butter cookies were added with catechins, curcumin, pectin and chitosan. The colors of cookies of different groups were represented by values of lightness (L*), redness (a*), and yellowness (b*) and were illustrated in Figure 5. As shown in Figure 5, cookies of the catechins group were obviously darker than the control group; this is not surprising because of the native color of catechins and their catechol or pyrogallol structures, which could stimulate browning during baking of the dough [46]. In addition, cookies of the curcumin group showed a yellow appearance (b* value range from 22–47) and were brighter than the control group. In fact, addition of catechins and curcumin brought an undesirable color to cookies, which negatively affected their acceptability. In contrast to the undesirable colors of catechins and the curcumin group, colors of cookie samples added with chitosan and pectin became lighter than the control group and the browning were obviously mitigated.

**Figure 6:**
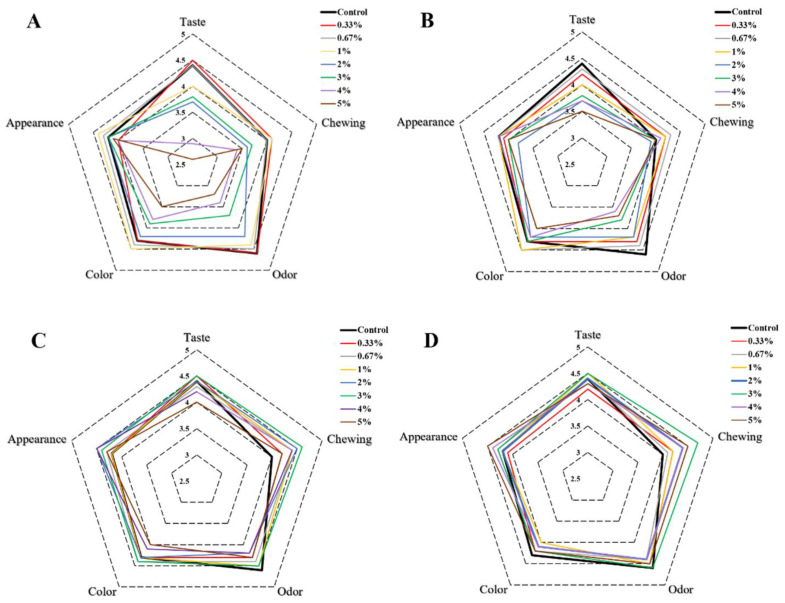
Radar plot of sensory results of different additions of cookies. ((**A**). catechins, (**B**). curcumin, (**C**). pectin, (**D**). chitosan).

The sensory qualities of different butter cookies were evaluated and the scores of sensory parameters were presented by a radar plot as shown in Figure 6A. The results suggested that the addition of natural antioxidants and hydrocolloids to butter cookies also had an impact on sensory qualities such as score of taste, chewing, odor, color and appearance. With an increase in the level of catechins (>1%), the cookies had a distinct and unpleasant astringency taste; thus, the taste values decreased significantly (*p* < 0.05). Similar changes occurred in the odor and color index, the aroma of cookies became weaker with more than 1% addition of CAT, and the color of cookies changed from light brown to dark brown, as described above. Curcumin also affected the sensory qualities such as taste, color and odor index, especially in terms of odor, cookies would smell slightly spicy (Figure 6B). Unlike natural antioxidants, the addition of hydrocolloids did not have an excessive impact on the sensory qualities. The color and the hardness of cookies would be slightly affected with the fortification of pectin and chitosan. The color would turn slightly lighter and the use of pectin and chitosan increased the hardness of cookies so that influenced the chewing score (Figure 6C,D).

The total sensory scores of cookies were calculated and were shown in Table 1. It is observed that the total sensory scores of cookies added with more than 2% of natural antioxidants were significantly decreased. For the pectin group, the total sensory scores would not decrease unless the adding level were more than 4%; besides, no significant differences in total sensory score were observed for the cookies spiked with different concentrations of chitosan (0.3–5%). Although chitosan had no obvious inhibitory effect on heat-induced AGEs and HMF, it has contributed to the sensory qualities of cookies, especially the chewing and appearance score. Therefore, combination of chitosan and catechins should be taken into consideration to inhibit AGEs and HMF in butter cookies without sacrificing of sensory qualities.

## 4. Conclusions

In summary, we evaluated the inhibitory effects of two dietary natural antioxidants (catechins and curcumin) and two dietary hydrocolloids (chitosan and pectin) against the formation of AGEs and HMF in butter cookies. The results indicated that catechins had appreciable inhibitory effects on AGEs and HMF formation, but pectin and chitosan are not ideal in their ability to simultaneously inhibit all heat-induced toxicants. In terms of analysis of color and sensory evaluation, compared with control group, the cookies manufactured with catechins and curcumin are not favored by consumers in terms of color and taste, and chitosan exerted the strong effect on improving the color and taste in the cookies.

In general, natural antioxidants showed an excellent inhibition on heat-induced toxicants, while hydrocolloids have good ability in maintaining or improving the taste and color of cookies. Between the necessary attributes of food sensory and healthier foods, we need to find a balance point to effectively control the heat-induced toxicants to a certain extent without destroying the food sensory. The combination of natural antioxidants with hydrocolloids may be a better choice.

## Figures and Tables

**Table 1:** Final total sensory score of cookies.

Additive	Control	Amount of Addition						
		0.3%	0.7%	1%	2%	3%	4%	5%
catechins	4.29 ± 0.24 ^a^	4.33 ± 0.24 ^a^	4.28 ± 0.23 ^a^	4.18 ± 0.22 ^a^	3.86 ± 0.20 ^b^	3.77 ± 0.22 ^b^	3.27 ± 0.24 ^c^	3.09 ± 0.17 ^c^
curcumin		4.21 ± 0.27 ^a^	4.30 ± 0.22 ^a^	4.15 ± 0.22 ^a^	3.84 ± 0.13 ^b^	3.88 ± 0.23 ^b^	3.84 ± 0.23 ^b^	3.78 ± 0.32 ^b^
pectin		4.34 ± 0.26 ^a^	4.34 ± 0.22 ^a^	4.35 ± 0.31 ^a^	4.30 ± 0.34 ^a^	4.47 ± 0.30 ^a^	4.23 ± 0.14 ^ab^	4.09 ± 0.32 ^b^
chitosan		4.23 ± 0.23 ^a^	4.29 ± 0.20 ^a^	4.24 ± 0.37 ^a^	4.33 ± 0.30 ^a^	4.48 ± 0.31 ^a^	4.31 ± 0.25 ^a^	4.34 ± 0.26 ^a^

Note: Values expressed are mean ± standard deviation (*n* = 3); means in the rows with different letters are significantly (*p* < 0.05) different.

## Data Availability

Not applicable.

## Supplementary Materials

The following are available online at https://www.mdpi.com/article/10.3390/foods11050657/s1, Table S1: Composition of the catechins, Table S2: The effects of chitosan and pectin on pH of dough.
