# Supplementary material for: Natural Antioxidants and Hydrocolloids as a Mitigation Strategy to Inhibit Advanced Glycation End Products (AGEs) and 5-Hydroxymethylfurfural (HMF) in Butter Cookies
Source: Foods. 2022 Feb 23;11(5):657. doi: 10.3390/foods11050657 (PMC8909267; doi:10.3390/foods11050657)
Supplement: Supplementary file 1 [file foods-11-00657-s001.zip › foods-1565493-supplementary.pdf]

Table S1 Composition of the catechins

	Of total (%)
Epigallocatechin	14.23
D-Catechin	1.61
Epicatechin	8.02
Epigallocatechin gallate	51.92
Gallocatechin gallate	1.95
Epicatechin gallate	14.10
Total Catechins	91.83

Table S2 The effects of chitosan and pectin on pH of dough

Dough		pH	Dough		pH
Control		8.37 ± 0.34 [a]			
	0.33%	8.35 ± 0.27 [a]		0.33%	8.45± 0.10 [a]
	0.67%	8.22 ± 0.15 [a]		0.67%	8.30 ± 0.08 [a]
	1.00%	7.97 ± 0.10 [ab]		1.00%	8.25 ± 0.24 [a]
Pectin	2.00%	7.70 ± 0.24 [b]	Chitosan	2.00%	8.47 ± 0.14 [a]
	3.00%	7.33 ± 0.15 [c]		3.00%	8.34 ± 0.27 [a]
	4.00%	6.87 ± 0.21 [d]		4.00%	8.22 ± 0.09 [a]
	5.00%	6.20 ± 0.37 [e]		5.00%	8.26 ± 0.12 [a]

[1] Values expressed are mean ± standard deviation (n = 3); means in the columns with different superscripts are significantly ($p < 0.05$) different.
